# The Yellow Fever

**Published:** 1871-05

**Authors:** 


### The Yellow Fever.

It seems that we are threatened with an epidemic of yellow fever this season, and it will require the greatest vigilance on the part of our various municipal Boards of Health to keep the disease from our shores. Advices from Buenos Ayres to April 12, have been received. The ravages of yellow fever were dreadful, and the deaths have increased to seven hundred per day.—*Med. and Surg. Reporter.*

;o:

—The method of watering streets with a solution of the chlorides of sodium calcium and aluminium, as proposed by Mr. Cooper, appears to have been successfully carried out in London.

